# TRAnexamic acid in hemorrhagic CESarean section (TRACES) randomized placebo controlled dose-ranging pharmacobiological ancillary trial: study protocol for a randomized controlled trial

**DOI:** 10.1186/s13063-017-2421-6

**Published:** 2018-03-01

**Authors:** Anne-Sophie Ducloy-Bouthors, Emmanuelle Jeanpierre, Imen Saidi, Anne-Sophie Baptiste, Elodie Simon, Damien Lannoy, Alain Duhamel, Delphine Allorge, Sophie Susen, Benjamin Hennart

**Affiliations:** 1 Pole anesthésie réanimation, maternité Jeanne de Flandre, centre hospitalier regional et universitaire, 2 avenue Oscar Lambret, 59037 Lille, France; 2 Laboratoire d’hémostase-hémobiologie, centre biologie pathologie, centre hospitalier regional et universitaire, 2 avenue Oscar Lambret, 59037 Lille, France; 3 Pharmacie centrale, centre biologie pathologie, centre hospitalier regional et universitaire, 2 avenue Oscar Lambret, 59037 Lille, France; 4 Unité de biostatistiques, Université Lille 2, 2 avenue Oscar Lambret, 59037 Lille, France; 5 Laboratoire de toxicologie, centre biologie pathologie, centre hospitalier regional et universitaire, 2 avenue Oscar Lambret, 59037 Lille, France

**Keywords:** Postpartum hemorrhage, Cesarean section, Fibrinolysis, Tranexamic acid, Pharmacokinetics, Plasmin, D-dimers

## Abstract

**Background:**

Evidence increases that a high or a standard dose of tranexamic acid (TA) reduces postpartum bleeding. The TRACES pharmacobiological substudy aims to establish a therapeutic strategy in hemorrhagic (H) Cesarean section (CS) with respect to the intensity of fibrinolysis by using innovative assays.

**Method/Design:**

The TRACES trial is a multicenter, randomized, double-blind, placebo-controlled, TA dose-ranging study that measures simultaneously plasmatic and uterine and urine TA concentrations and the plasmin peak inhibition tested by a simultaneous thrombin plasmin generation assay described by Van Geffen (novel hemostasis assay [NHA]). Patients undergoing H CS (>800 mL) will receive blindly TA 0.5 g or 1 g or placebo. A non-hemorrhagic (NH) group will be recruited to establish plasmin generation profile. Venous blood will be sampled before, at the end, and then at 30, 60, 120, and 360 min after injection. Uterine bleeding will be sampled after injection. Urine will be sampled 2 h and 6 h after injection. The number of patients entered into the study will be 114 H + 48 NH out of the 390 patients of the TRACES clinical trial.

**Discussion:**

To explore the two innovative assays, a preliminary pilot study was conducted. Blood samples were performed repeatedly in patients undergoing either a H (>800 mL) or NH (<800 mL) CS and in non-pregnant women (NP). H patients received TA (0–2 g). Dose-dependent TA plasmatic concentrations were determined by LC-MS/MS quantification. Plasmin generation and its inhibition were tested in vitro and in vivo using the simultaneous thrombin–plasmin generation assay (STPGA). The pilot study included 15 patients in the H group, ten patients in the NH group, and seven patients in the NP group. TA plasmatic concentration showed a dose-dependent variation. STPGA inter-assay variation coefficients were < 20% for all plasmin parameters. Inter-individual dispersion of plasmin generation capacity was higher in H and NH groups than in NP group. Profile evolution over time was different between groups. This preliminary technical validation study allows TRACES pharmacobiological trial to be conducted.

**Trial registration:**

ClinicalTrials.gov, NCT02797119. Registered on 13 June 2016.

**Electronic supplementary material:**

The online version of this article (10.1186/s13063-017-2421-6) contains supplementary material, which is available to authorized users.

## Background

Postpartum hemorrhage (PPH) is the leading cause of maternal death. Tranexamic acid (TA) (Exacyl®, Sanofi, France), an antifibrinolytic drug, reduces bleeding and transfusion need in major surgery and trauma [1, 2]. In ongoing PPH following vaginal delivery, a high dose of TA decreased PPH volume and duration and maternal morbidity, while early fibrinolysis was inhibited [3, 4]. In a large international, placebo-controlled, randomized controlled trial (RCT) using a uniform unique 1-g dose, TA reduced maternal mortality due to bleeding but did not succeed in reducing the hysterectomy rate or the global rate of mortality [5]. Despite the high mortality due to recruitment in low resources countries, TA avoided 150 deaths out of the 10,051 treated patients compared to the 10,009 placebo patients. This beneficial impact occurred when TA was administered 1–3 h after PPH onset [5]. TA efficiency and optimal dose in hemorrhagic (H) CS has to be determined. A dose-ranging study is needed to assess the optimal TA dose to be administered. Pharmacokinetics studies conducted in the 1980s identified TA (p-aminomethyl cyclohexane carboxylic acid [AMCHA]) in its trans-form (AMCA) as 6–10 times more potent than its precursor epsilon aminocaproic acid (EACA) [6–12]. In healthy non-hemorrhagic (NH) volunteers, doses of 2.5–100 mg/kg were tested intravenously and orally [7–11]. More recently, well conducted pharmacokinetics studies have been performed after TA administration in adult [13, 14] and pediatric [15–18] cardiac surgery. Studies used a single bolus in the range of 10–100 mg/kg. Others used repeated boluses or a continuous infusion during and/or after cardiopulmonary bypass. A minimal concentration able to prevent in vitro-induced fibrinolysis has been evaluated and seems lower in neonates than in adults [19]. Several factors may explain the high degree of heterogeneity observed among these studies [14–18]. These first results using concomitant clinical and pharmacological effects evaluation suggest that the higher doses used in cardiac surgery, and considered as efficient, could be reduced [17]. This is of interest regarding the side effects (seizures and visual disturbances) that seem more frequent in higher doses series [13, 20–23]. On the contrary, the Crash2 study in trauma using a unique dose of 1 g demonstrated the absence of excessive thrombotic or renal failure in the TA-treated group [21]. In active PPH, nausea and visual disturbances were also noted after the higher dose of 4 g then 1 g per hour over 6 h. No excessive risk of renal failure and deep vein thrombosis were observed [24].

### PPH-induced coagulopathy and TA-related fibrinolysis inhibition in obstetrics setting

**Fig. 1:**
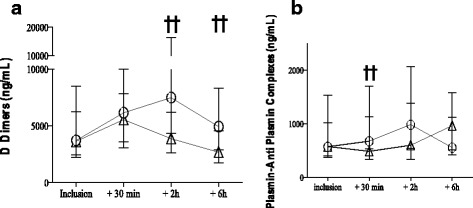
Inhibition of the natural hyperfibrinolysis by a high dose of TA administered at the early stage of PPH. D-Dimers and PAP complex evolution at the onset of PPH and 30, 120, and 360 min later in the untreated H group compared to the TA-treated group

Fibrinolysis inhibition has been observed biologically while comparing TA treated haemorrhagic group to non treated haemorrhagic group or non haemorrhagic postpartum [4]. Significantly excessive fibrinolysis was noted in the untreated H group vs the NH group since the PPH onset. This excessive fibrinolysis was inhibited in the TA treated group 30 min and 2 h after TA administration, as demonstrated by the inhibition of the D-dimers and plasmin antiplasmin (PAP) complex increases (Fig. 1) [4].

**Fig. 2:**
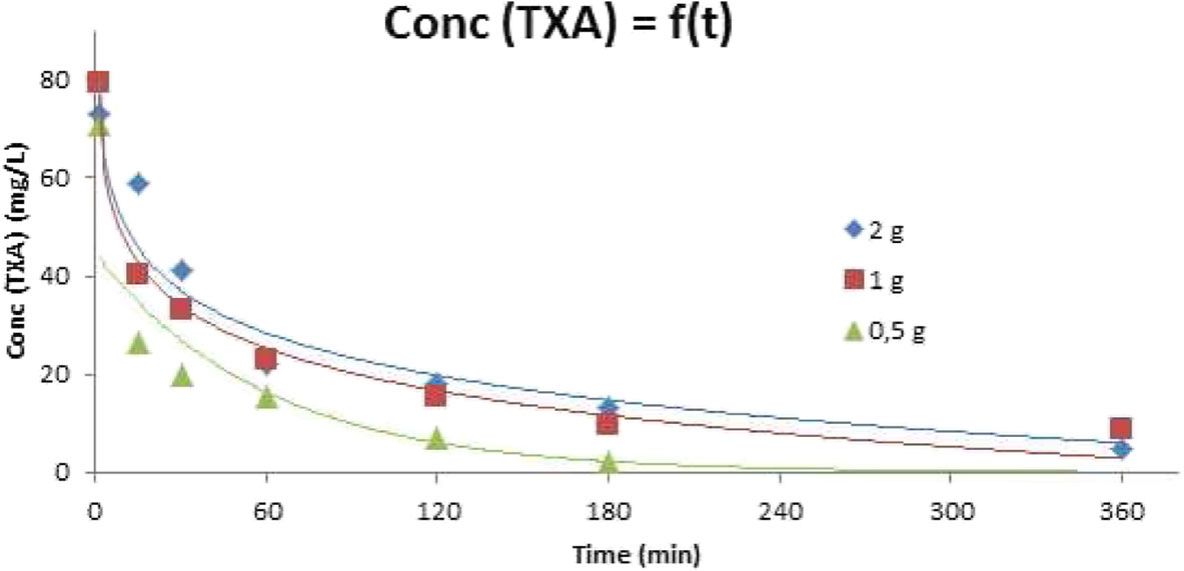
TA plasma concentration measurement method: determination of the optimal timing. A timed urinary sample is collected in a graduated urinary bag between inclusion (T0) and T120, then T120 and T360, and end of the observation period of 6 h to measure the cumulative dose of TA excreted in urine

**Fig. 3:**
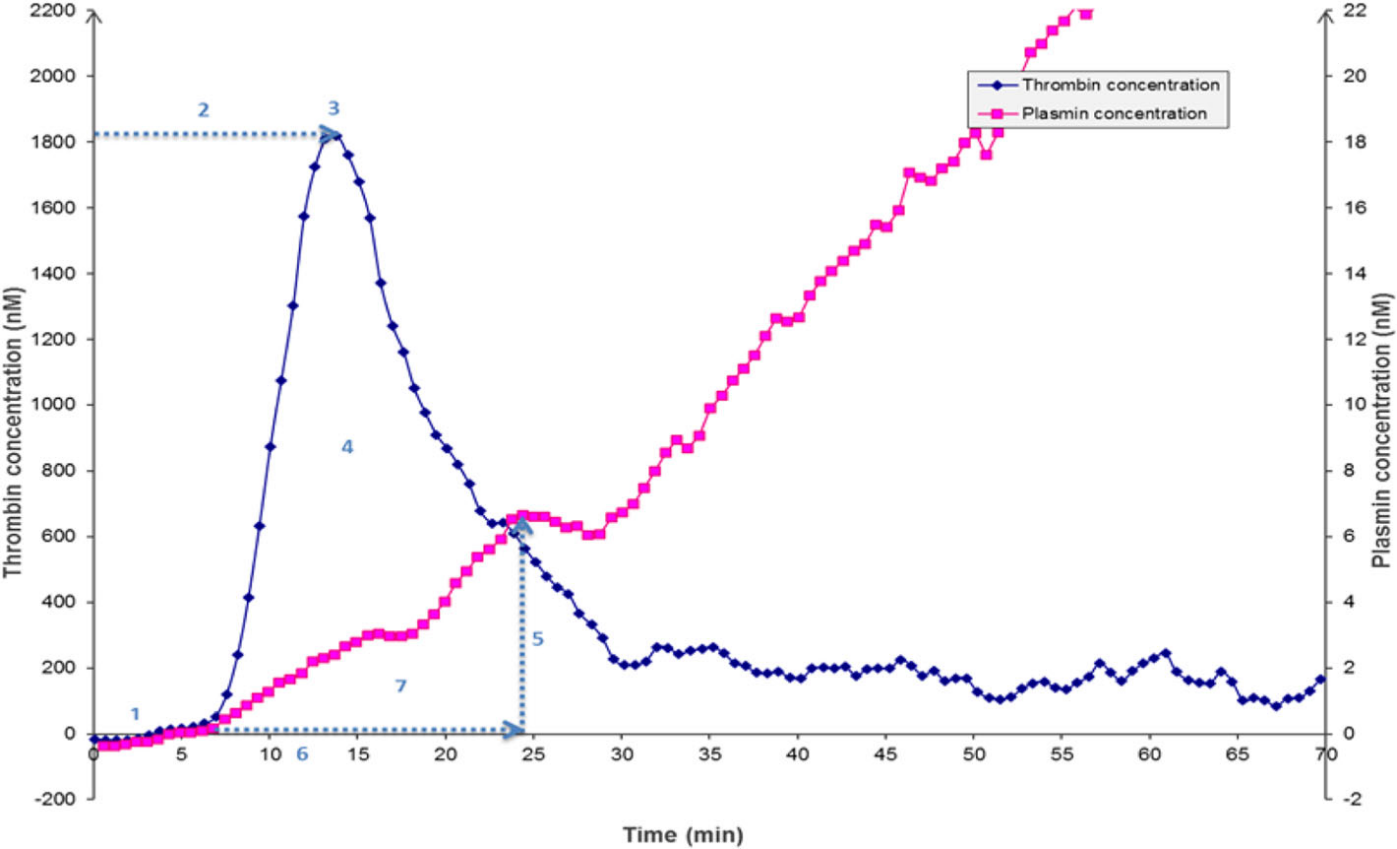
Parameters studied in the simultaneous thrombin–plasmin generation assay. Thrombin parameters: (1) lag time (min); (2) thrombin peak time (min); (3) thrombin peak (nM); and (4) area under the curve (AUC) (nM.min). Plasmin parameters: (5) plasmin peak (nM); (6) fibrin lysis time (FLT) (min); and (7) AUC during FLT (plasmin potential) (nM.min)

Fibrinogen decrease is a central component of PPH-induced coagulopathy [4, 25, 26]. Fibrinogenolysis is induced by two potential enzymes: thrombin and plasmin. Thrombin divides fibrinogen to fibrin monomers, which are then stabilized to fibrin clot by activated factor XIII. Fibrin formation is the final step of plasmatic coagulation. Plasminogen links to native fibrin in a tertiary complex tPA-fibrin-activated plasminogen (i.e. plasmin). Plasmin divides stabilized fibrin to D-dimers and plasmin in excess divides fibrinogen and factor V directly.

In PPH due to uterine atony after vaginal delivery, fibrinogen decrease is an active coagulopathic process as the amplitude of the decrease is greater than the one expected after dilution or H loss [26]. Active fibrinogenolysis remains a major component in amniotic fluid embolism, fetal death, or placenta abruptio [27–29]. Concomitant D-dimers increase and factor II decrease are active and early phenomena. TA was efficient in reducing D-dimers and PAP complexes peak [4] as well as in placenta abruptio, but had no influence on fibrinogen decrease [24, 29].

### TRACES study is an opportunity to describe the pharmacobiological profile of two doses of TA in hemorrhagic Cesarean section

A multicenter randomized double-blind placebo-controlled dose-ranging study, TRACES, is ongoing to demonstrate the relative efficacy and safety of a low or standard dose of TA compared to placebo (article princeps). The objective of this therapeutic study is to measure the effect on blood loss reduction of a single intravenous infusion of two TA doses regimens administered at the onset of an active PPH (>800 mL) during non-emergent Cesarean section (CS).

As a substudy of the TRACES trial, a pharmacobiological profile will be performed to correlate the clinical effect with the biological fibrinolysis inhibition and establish a pharmacokinetics model regarding the TA plasma and uterine bleeding concentration. to prepare the pharmacobiological part of TRACES study, a TRACES pilot study was conducted to evaluate the feasibility, accuracy, and variation coefficient of both the drug concentration measurement technics and the simultaneous thrombin and plasmin generation assay (STPGA).

The present article describes the method and preliminary results of the TRACES pharmacobiological substudy and its preliminary pilot study for technical validation of the biological methods.

## TRACES pharmacobiological substudy

### Method and design

#### Experimental plan and ethics authorization

The TRACES trial is a multicenter double blind randomized trial approved by the north and west of France ethics committee (15/50_020216). The TRACES pharmacobiological substudy is an ancillary study of the TRACES trial (Additional files 1 and 2).

#### Experimental plan

**Fig. 4:**
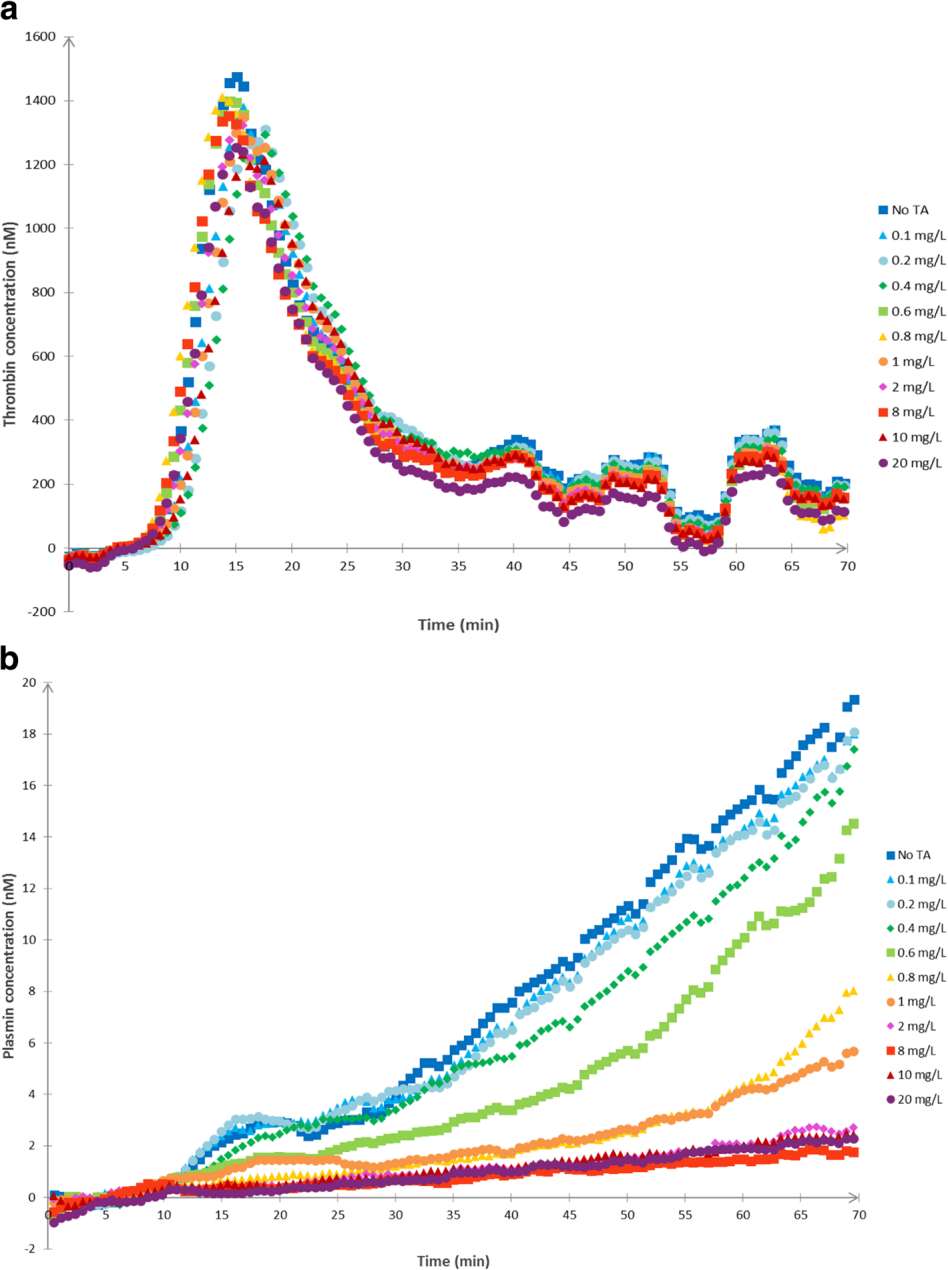
**a** In vitro STPGA with different TA concentrations: thrombin generation. In vitro dose effect relationship between TA and thrombin generation. Thrombin generation in the STPGA performed with a pooled plasma sample and different TA concentrations in the range of 0–20 mg/L. **b** In vitro STPGA with different TA concentrations: plasmin generation. In vitro dose effect relationship between TA and plasmin generation. Plasmin generation in the STPGA performed with a pooled plasma sample and different TA concentrations in the range of 0–20 mg/L

TRACES study is a multicenter, randomized, double blinded, placebo controlled therapeutic and pharmacobiological dose ranging study to evaluate the clinical efficiency of TA on blood loss reduction in patients experiencing PPH during elective or non-emergent CS (Fig. 4).

**Fig. 5:**
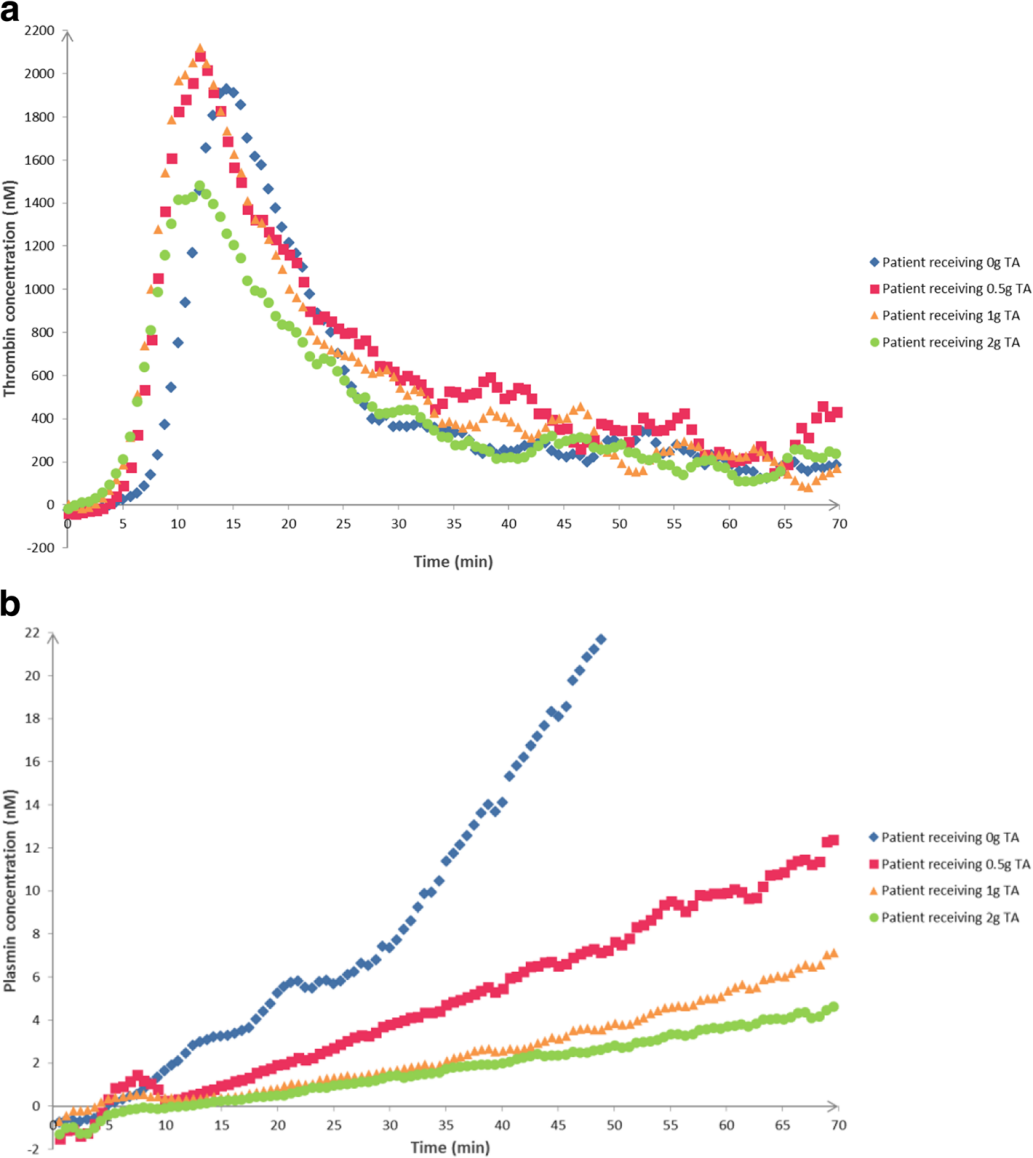
**a** In vivo study: TA dose impact on thrombin generation profiles at T30. In vivo thrombin generation variations at T30 depending on TA dose administered: four patients who received 0, 0.5, 1, and 2 g (IV bolus), respectively. **b** In vivo study: TA dose impact on plasmin generation profiles at T30. In vivo plasmin generation variations at T30 depending on TA dose administered: four patients who received 0, 0.5, 1, and 2 g (IV bolus), respectively.

**Fig. 6:**
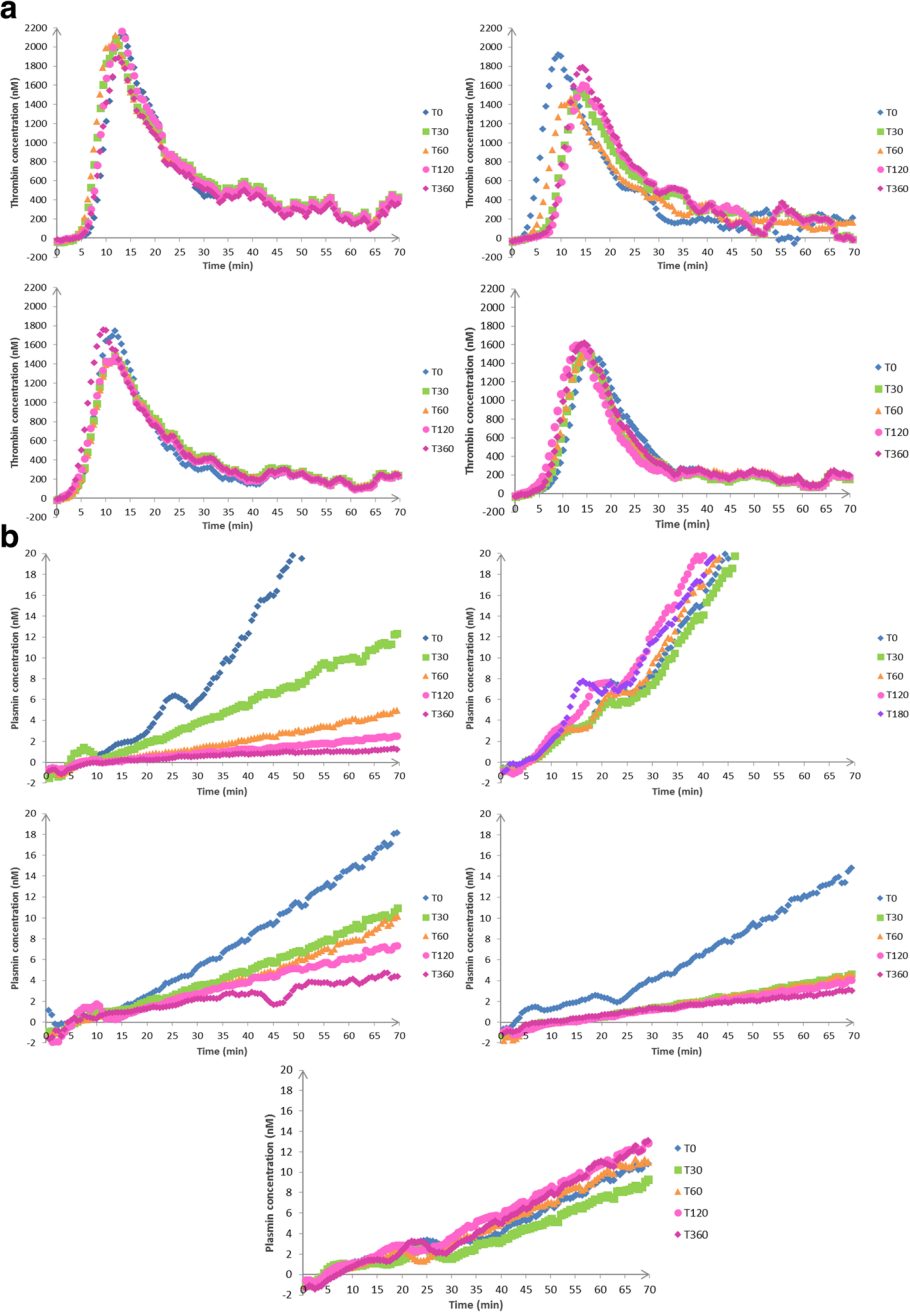
Time repeated profiles: evolution of thrombin (**a**) and plasmin (**b**) profiles over time points: T0, T30, T60, T120, and T360, in patients in the NH group, the H group, and TA group with increasing doses of TA: TA1/2: 0.5 g, TA1: 1 g, and TA2: 2 g. A-NH evolution of thrombin generation profile over time for a NH patient, B-NH evolution of plasmin generation profile over time for a NH patient, A-0 evolution of thrombin generation profile over time for a H patient receiving 0 g TA, B-0 evolution of plasmin generation profile over time for a H patient receiving 0 g TA, A-TA1/2 evolution of thrombin generation profile over time for a H patient receiving 0.5 g TA, B-TA1/2 evolution of plasmin generation profile over time for a H patient receiving 0.5 g TA, A-1 evolution of thrombin generation profile over time for a H patient receiving 1 g TA, B-1 evolution of plasmin generation profile over time for a H patient receiving 1 g TA, A-2 evolution of thrombin generation profile over time for a H patient receiving 2 g TA, B-2 evolution of plasmin generation profile over time for a H patient receiving 2 g TA

#### Participating centers involved in the research program

The five French academic medical centers (Lille, Valenciennes, Lyon Croix Rousse, Paris Louis Mourier, Paris Trousseau) are known for their strict multidisciplinary management of PPH, including systematic measurement of blood loss, and for their ability to perform research programs. Anesthetic and obstetric staff at each center agreed to participate in this study and were involved in writing the French guidelines for PPH management [22]. The study will be sponsored by The University and Regional Hospital Centre of Lille, France.

#### Study population

The inclusion criteria for the experimental group concern each patient experiencing a bleeding volume of ≥ 800 mL due to surgery or to uterine atony during an elective or non-emergent CS. The NH group, where patients experience a bleeding volume < 800 mL, will be sampled to obtain a reference profile for the direct plasmin generation test in CS. All patients receive complete information, give their written consent and benefit from national social security.

Non-inclusion criteria are as follows: hypersensitivity to the product or its excipients; previous or ongoing arterial or venous thrombosis; disseminated intravascular coagulopathy, except disseminated intravascular coagulopathy (DIC) associated with a predominant fibrinolytic profile; renal failure; previous seizures; severe HELLP syndrome; emergent CS; administration of TA before inclusion; inherited H diseases and low molecular weight heparin (LMWH) within 24 h before inclusion; previous inclusion in an interventional trial for two months; or patient unable to give consent.

### Study protocol

#### Screening phase: information to participants

During the routine third trimester pre-anesthetic assessment, every patient is informed about research programs focusing on PPH and TA in H CS. During the pre-anesthetic visit, inclusion and non-inclusion criteria are screened; fully detailed information including information on pharmacobiological substudy is given. Informed consent is signed before Cesarean onset.

#### Randomization, study drug production, and blinding and TA stability assessment

##### Description of the unit dose, packaging, and labeling of the study drug

The doses are packaged by the pharmacy of the sponsor in a blind way for every investigator. Each package is numbered following the randomization table established by center. The package includes syringes or flacons of 10 mL of the studied product. The preparation of the product in each group is TA1 (10 mL = 1 g TA + 0 mL saline), TA½ (5 mL = 0.5 g TA + 5 mL saline) or TA0 (0 mL = 0 g TA + 10 mL saline).

##### Compliance to the drug administration is checked during the follow-up visits

ᅟ

##### Methods of storing the investigational drug

The preparation of 10 mL study-syringes is achieved by the pharmacy of the sponsor in numbered boxes depending of the randomization by center. Every syringe and kit are labelled according to the current regulation. The boxes are allocated to the centers via their pharmacy research unit and five boxes are available in the labor ward in order to answer to elective or emergent inclusion processes. The product is allocated to the patient following an increasing number. Boxes are returned to the center pharmacy to be traced.

##### Stability study and description of the study drug production

To maintain the blindness during the TRACES trial, pharmacists are solicited to prepare ready-for-use vials containing TA (Exacyl® 0.5 g/5 mL, Sanofi-Aventis, France) at 1 g/10 mL or 0.5 g/10 mL or placebo. As the stability of TA in vials at these concentrations has, to our knowledge, not been previously studied, a preliminary study was performed by the toxicology laboratory of the Lille center and confirmed the stability of TA in glass vials up to one year.

### Blind randomized treatment administration

The preparation of the two dose-regimens and the placebo is planned for each center by the promoter to certify the double-blind character of the trial. The products are prepared as ready to use.

The treatment is presented in a blinded 10-mL syringe containing 0, 0.5, or 1 g TA, respectively, for each TA0 (placebo), TA½, and TA1 group. The single-dose injection is independent from patient weight in order to allow a pharmacokinetic analysis of the weight influence. Treatment administration is performed after birth.

The time of injection onset is noted as T0. Intravenous injection is performed with a strict control of 1-min duration and the end of injection defines T1, which corresponds to the plasmatic concentration peak of TA.

Rescue administration of a second dose of 1 g TA is allowed only if hemorrhage becomes severe (>1500 mL). If this condition cannot be respected, the patient is excluded a posteriori. The inclusion number is kept for the patient presenting this deviation to the protocol; the follow-up and visits are continued and analyzed in the intention-to-treat (ITT) analysis.

The investigator can use, at any moment and in any situation that seems necessary, the unblinding procedure in using envelopes attached to the treatment.

### PPH management and care standardization

The uterotonic treatment, vascular loading, and transfusion follow the French PPH guidelines, as described in the following paragraph. Uterotonic treatment strictly respects the guidelines for PPH prevention: systematic prophylactic infusion of oxytocin by electric syringe (10 UI in 10 min, then 10 UI over 60 min, then 10 UI over 5 h). A 5 UI intravenous bolus and a 20 UI/20-min infusion are administered in cases of atony. Sulprostone or prostaglandin treatment: 500 μg in 1 h, then 500 μg over 6 h, is initiated in cases of oxytocin inefficiency.

Vascular loading use gelatin or crystalloids compensates exact blood loss volume > 800 mL. The criteria for colloid adjustment and hemodynamic assessment are the reduction of tachycardia to < 100 bpm, the stability of diuresis volume at > 40 mL per hour, and a systolic blood pressure > 80 mmHg. The non-invasive hemodynamic device NEXFIN® (Edwards, Philadelphia, PA, USA) should be used for this assessment. Red blood cell transfusion is strictly conducted regarding guidelines to maintain the hemoglobin level at > 8 g/dL. The follow-up of the hemoglobin level use delocalized devices such as repeated hemocue® or continuous hemoglobin monitoring Massimo® France. Cell salvage is used following the international guidelines. Fibrinogen concentrates can be used in association with TA when Clauss-plasma fibrinogen is < 2 g/dL and A5 FIBTEM < 8 mm or MCF FIBTEM < 20 mm. Prothrombin complex concentrates, factor XIII or recombinant factor VIIa can be discussed in cases of hysterectomy. Obstetrical rescue invasive procedures follow the French guidelines for PPH management in cases of persistent massive hemorrhage: intrauterine compression balloon; embolization or surgical arterial ligatures; and surgical compression technics. Hysterectomy is considered for maternal rescue.

### Assessments

#### Efficacy assessment: blood loss measurement

Blood loss volume (mL) is measured in the surgical or cell saver aspiration bag, in the delivery bag collecting vaginal blood flow during CS, and by weighing drapes and pads. Antiseptic and amniotic fluids must be strictly separated, counted, and subtracted.

A double assessment of the primary criterion using hemoglobin (Hb) drop-based blood loss calculation is performed. Our secondary criterion aims to promote scientific data for potential dose reduction and the potential of reduced maternal morbidity.

#### Safety assessments

Side effects are recorded at each study time. Side effects assessment consists of seizures and visual disturbances, renal failure, and nausea and vomiting. Thromboembolism and any organ failure are recorded.

#### Data collection

Inclusions are performed 24 h per day, seven days a week. Each patient admitted for an elective or non-emergent CS during labor is informed before the beginning of the CS and a signed consent is collected. Patients are included in the H group when hemorrhage > 800 mL. The blind injection of the 10-mL vial containing 0, 0.5, or 1 g of TA is performed over 1 min between T0 and T1 after injection. Blood loss, transfusion, maternal hemodynamics, resuscitation measures, uterotonic and invasive procedure data, and maternal morbidity parameters are recorded before inclusion (T0) and at T30, T60, T120, T360 (30, 60, 120, and 360 min, respectively, after inclusion). A follow-up visit collecting total blood loss and maternal morbidity takes place at day 2 (±12 h) and at day 42 (±14 days) postpartum during a phone interview.

Various margins of time will be allowed for the timing of the data collection: T1 is the reference time and the time of the TA plasmatic concentration peak and no margin will be allowed (sample exactly at the end of the TA injection). The margins allowed for T30, T60, T120, and T360 are 5, 5, 10, and 60 min, respectively. The margins allowed for day 2 are 12 h and for day 42 14 days. Real-time data collection is carried out to show the pharmacodynamic analysis.

#### Biological assessment

##### Preanalytical management

Tubes are prepared in advance by the local investigator. Samples are identified by colored codes. Samples are brought to the laboratory via a short circuit and current biological parameters of coagulation, blood count, and renal function are performed on site and communicated directly to the clinician in charge of the patient. Concerning the TA concentration analyses and STPGA, as well as any future contributive biological method to diagnose and treat fibrinolysis, plasmas will be separated by centrifugation (2500 g × 15 min at 20 °C), then collected and frozen to be kept at – 70 °C in each center in a box with the inclusion number and the time of sampling to identify the sample. At the end of the inclusions, all plasmas will be transferred to the Biology and Pathology center of the Lille University Hospital in dry ice by an agreed carrier (authorized L1243-4 in French Public Health Code).

##### Non-specific biological assessment

Blood for laboratory tests is sampled using the second venous catheter currently placed as a safety procedure for CS surgery at T0 and day 2. Laboratory non-specific tests are carried out in each center using guidelines usual rules and devices. The reference values are collected. Laboratory non-specific tests may include complete blood count with platelet count, coagulation screening including aPTT, PT, fibrinogen, factors II and V, antithrombin, fibrin monomers, and D-dimers, as well as renal function parameters. Complete blood count is measured on the morning at day 2 after CS to obtain the comparison with the third trimester results.

##### Specific biological assessment

**Fig. 7:**
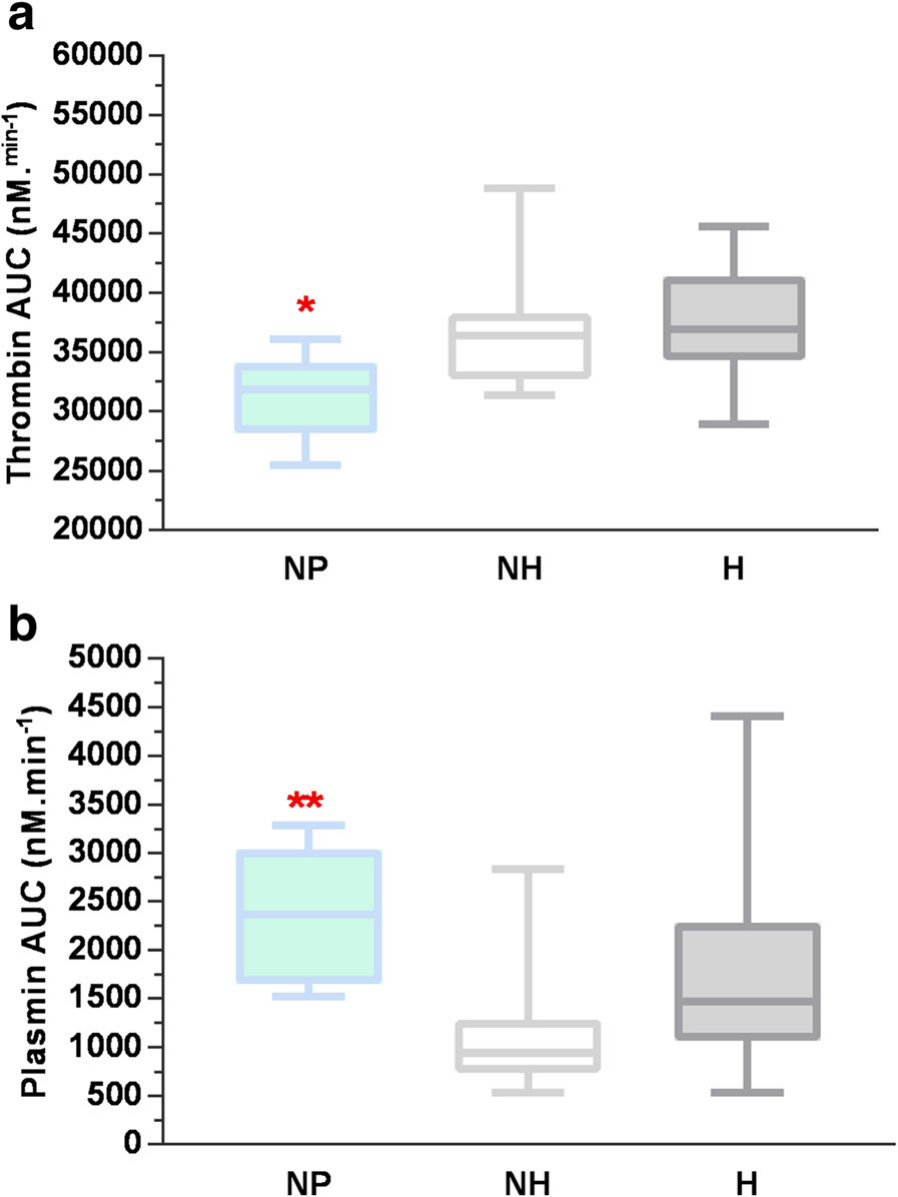
Thrombin (**a**) and plasmin (**b**) AUCs measured in STPGA at T0 in NH and H + TA groups compared to the NP group. AUC area under the curve, NP non-pregnant group, NH non-hemorrhagic group, H hemorrhagic untreated group, TA hemorrhagic treated group. *Thrombin AUC in NP vs NH group: *p* = 0.02; **Plasmin AUC in NP vs NH group: *p* = 0.002

A pharmacobiological substudy (Fig. 7) is supported by the French National Agency for Medicine and Health Products research program. Specific venous blood samples are made as part of the biological sampling before inclusion and at follow-up visits at T0, T30, T60, T120, and T360. Two specific tests are planned: simultaneous thrombin–plasmin generation assay in a well (STPGA) [30] and determination of TA concentration [31]. Both of these innovative tests will be performed on a venous blood and a uterine bleeding sample in order to draw a pharmacokinetic distribution of the product and its effect on fibrinolysis. The TA concentration analysis will be performed only on patients in the H group. A specific sample of uterine bleeding poured out intraoperatively is performed at T0/T1 and T15 and as long as the uterine bleeding continues. A T0–T120 and T120–T360 timed urinary sample is collected in a graduated urinary bag to measure the TA urinary cumulative excretion.

### Specific management of the ancillary pharmacobiological study

#### Inclusion criteria

Out of the 342 H patients included in the TRACES trial (CS with bleeding > 800 mL), a subgroup of 144 patients will be included in the substudy, for which specific (SGTPA and TA concentration) biological samples and analysis will be collected. The criteria for the inclusion in the substudy are organizational criteria: the sampling and the management of plasma aliquots need daytime conditions. Each patient included in the TRACES trial in these conditions may be included in the TRACES substudy.

The 48 NH group (CS with bleeding < 800 mL) inclusions are all expected to be included in the TRACES pharmacobiological substudy to obtain the reference data for the SGTPA.

#### Exclusion criteria

Patients not included in TRACES trial or for which the sampling is difficult (difficult venous access or severe sudden hypovolemia).

#### Individual and global risk-benefit of the substudy inclusion

There is no individual risk or benefit for the patient except the supplementary sampling of two tubes of 4 mL and one tube of 2 mL five times in the H group and two tubes of 4 mL in the NH group. As a benefit for the scientific knowledge, the TRACES substudy will furnish the first data on dose ranging pharmacokinetics of TA in H CS.

### Trial conduct and data monitoring

#### Study duration

Enrolment period: 2016–2018.

For a given individual: 42-day participation after randomization.

Duration of research: 44 months.

End of the research term following participation of the last enrolled person: 1 year.

#### Data collection and management

Data are confidential, in accordance with the law dated 6 January 1978. Data are collected electronically. Data will be analyzed in accordance with methodology described in the MR 06001 form of the French data protection authority by Pr Alain Duhamel’s epidemiology and public health biostatistics platform Lille University Hospital Lille. Access to data will be restricted to individuals who are directly involved in the study. Data may be modified by any physician participating in the study or a fellow working with a physician participating in the study. Trial data will be archived for at least 15 years after the trial has ended.

### Study outcomes

#### Primary endpoint

The primary endpoint of the TRACES trial is the additional blood loss volume measured between inclusion (T0) and 6 h after inclusion (T360).

#### Endpoints of the substudy

The secondary biological endpoints measure the impact of the study product and dose on D-dimers level and hemostasis parameters, specifically focusing on the diagnosis and evolution of fibrinolysis, plasmin generation, and plasma and uterine TA concentrations in order to create a pharmacokinetic model of TA doses in H CS.

### Trial conduct and data monitoring

#### Study duration

Enrolment period: 2016–2018.

For a given individual: 42-day participation after randomization.

Duration of research: 44 months.

End of the research term following participation of the last enrolled person: 1 year.

#### Data collection and management

Data will be anonymized in accordance with the law dated 6 January 1978. Data will be collected electronically. Data will be analyzed in accordance with the methodology described in the MR 06001 form of the French data protection authority by Pr Alain Duhamel’s epidemiology and public health biostatistics platform Lille University Hospital Lille. Access to data will be restricted to individuals who are directly involved in the study. Data may be modified by any physician participating in the study or a fellow working with a physician participating in the study. Trial data will be archived for at least 15 years after the trial has ended.

### Statistical method

#### Sample size calculation

The sample size computation is based on the expected difference between the placebo group and the low dose and available data on TA use in active PPH.

Given that there is no available data on low TA dosage impact on blood loss reduction after inclusion, and considering a 50% reduction as previously observed after a higher dose administration, the required number of participants is 103 per group for a type I error of 5% and a power of 80%. Considering a maximum of 10% of drop-outs or missing data, we will recruit 114 H patients per group for a total of 342 patients.

In order to compare the specific fibrinolysis inhibition parameters with the recent thrombin–plasmin potential generation in a well, a subgroup of patients are sampled for these specific biological tests and a complementary group of non-treated, NH CS patients will be included in an observational sequence of clinical and biological assessments. The number needed to observe these pharmacobiological secondary objectives has been calculated regarding the inhibition of fibrinolysis as diagnosed by the number of patients for whom D-dimer level increase between 30 and 120 min was negative or null (EXADELI trial [21]). The number necessary to treat for this substudy is 48 patients in each of the three H groups and 48 patients in the reference NH group, for a total of 192 patients. These substudy patients will be selected out of the 342 patients of the experimental groups and recruited at the time when sampling, collection, and freezing are available.

#### Statistical analysis

Statistical analyses will be independently performed by the Biostatistics Department of University of Lille under the responsibility of Pr. Alain Duhamel. Data will be analyzed using the SAS software (SAS Institute Inc, Cary, NC, USA) and all statistical tests will be performed with a two-tailed alpha risk of 0.05. A detailed statistical analysis plan will be written and finalized before the database lock. Baseline characteristics will be described for each group. The quantitative variables will be expressed as mean and standard deviation in cases of normal distribution and median and interquartile if not. The normality of distributions will be checked graphically using the Shapiro–Wilk test. Qualitative variables will be expressed as the frequencies and percentages.

For the main objective, the blood loss measured in each experimental group (low dose and high dose) will be compared to that of the placebo group by using an analysis of covariance adjusted for baseline blood loss volume and center-effect. In cases of non-normal distribution (except if a log-transformation could be applied to normalize the data), relative blood loss volume will be calculated and compared using a Mann–Whitney U test.

Analyses will be done on an ITT basis, especially concerning the patients receiving a rescue dose of TA in cases of severe (>1500 mL) hemorrhage. An exploratory analysis will be performed on all the measures of the primary endpoint (T0, T30, T60, T120, T360). We will use the linear mixed model to compare the evolution of the primary endpoint according to each experimental group and the placebo group. This model allows performing an ANOVA test for repeated measures taking into account the correlation between the repeated measures. The choice of the covariance model will be based on the AIC criteria. Post hoc analysis at each time of measure will be performed using a Bonferroni correction.

For the secondary objectives, secondary endpoints will be compared between each experimental group and the placebo group by using the Chi-square test or Fisher’s exact test for qualitative parameters and using Student’s t-test or Mann–Whitney U-test for quantitative parameters. For the quantitative parameters measured at each time point, we will employ the linear mixed model, as previously described for the main objective. The incidence of adverse events will be analyzed in a descriptive way.

## Technical validation of the biological method for TA dosage and simultaneous generation thrombin plasmin assay (SGTPA)

Innovative simultaneous thrombin plasmin generation assay and the TA concentration measurement require a technical validation. That is why a monocentric descriptive pilot study has been performed and is described in the following chapter.

### Pilot study design

#### Inclusion criteria

Patients undergoing CS, either NH (blood loss < 800 mL) or H (blood loss > 800 mL), receiving various doses of TA in the range of 0–2 g as recommended in the North of France perinatal net PPH management algorithm.

#### Exclusion criteria

Non-inclusion criteria are as follows: hypersensitivity to the product or its excipients; previous or ongoing arterial or venous thrombosis; disseminated intravascular coagulopathy, except disseminated intravascular coagulopathy (DIC) associated with a predominant fibrinolytic profile, renal failure; previous seizures; severe HELLP syndrome; emergent CS; administration of TA before inclusion; inherited H diseases and LMWH within 24 h before inclusion; previous inclusion in an interventional trial for two months; or patient unable to consent.

#### PPH management

PPH management was conducted following the French and North of France perinatal net guidelines especially for the uterotonics administration, vascular loading, and transfusion.

### Pilot study conduct

#### Patient screening

Each patient undergoing a non-emergent CS received the information and signed consent before the beginning of the CS. Anthropomorphic, anesthetic, and obstetrical data were collected.

#### Inclusion

Inclusion was performed 10 min after delivery in NH CS and at the time of 800 mL bleeding in H CS. TA current dose of 0, 0.5, 1, or 2 g were administered intravenously over 1 min following the guidelines. The specific venous blood samples were performed as a part of the current biological sampling on a dedicated venous catheter set up for the surgical procedure.

#### Timing of the biological samples

The timing of sampling was T0 (inclusion time when bleeding ≥ 800 mL was diagnosed), T1 (at the end of injection), and T15, T30, T60, T120, T180, and T360 (defined as 15, 30, 60, 120, 180, and 360 min later, respectively). A timed urinary sample was collected in a graduated urinary bag between inclusion (T0) and T120 then T120 and T360, at the end of the observation period of 6 h to measure the cumulative dose of TA excreted in urine.

Various timing margins have been allowed for samples’ collection: T1 is the reference time and the time of the TA plasmatic concentration peak and no margin will be allowed (sample exactly at the end of the TA injection), The margins allowed for T30, T60, T120, T360 were 5, 5, 10, and 60 min, respectively. The exact timing of the data collection was noted to show the pharmacodynamic analysis.

Usual non-specific laboratory tests were carried out using the usual rules and devices. Laboratory non-specific tests included complete blood count with platelet count, coagulation screening including aPTT, PT, fibrinogen, factors II and V, fibrin monomers and D-dimers, PAP complexes, and renal function.

Tubes used to collect blood samples were prepared ahead of time by the local investigator and identified by color codes. Samples were brought to the laboratory in a short circuit and routine biological parameters of coagulation, blood count, and renal function were performed on site and communicated directly to the clinician in charge of the patient. Regarding the innovative biological methodology for TA concentration and STPGA or any future contributive technic to diagnose and treat fibrinolysis, plasmas were separated by centrifugation (2500 g × 15 min at 20 °C) then collected and frozen to be kept at – 80 °C in Centre of Biologie Pathologie CHRU de Lille.

### Objectives

The primary objective was to measure the variation coefficient of SGTPA for each generation: thrombin and plasmin.

Secondary objectives were: to confirm the feasibility of the trial channels for sampling distribution, the reliability of TA concentration measurement method, and the feasibility of the SGTPA and TA concentrations relationship models.

### Statistical method

Clinical and biological data were collected using Excel® (Microsoft). The number needed to study in this pilot study was estimated to ten patients in the H group and ten patients in the NH groups. Non-pregnant (NP) values were extracted out of a previous validation study.

Qualitative variables were analyzed regarding frequency and percent as quantitative variables were described by median and interquartile or mean and standard deviation. Comparison used non-parametric tests (Mann–Whitney and Kruskal–Wallis tests) and Student’s t-test for NH and H mean values. Correlation was evaluated by Spearman coefficient. *P* < 0.05 was considered significant. Statistical analyses and graphs were performed using GraphPad Prism® (version 7).

### TRACES pilot study: results

#### Population

The TRACES non-interventional pilot study was conducted in the North of France academic hospital center over eight months. Parallel data from a NP group of healthy women of childbearing age (n = 7) were available. Patients were recruited after informed consent and distributed in three groups defined as follows: patients experiencing a NH CS with a total bleeding < 800 mL group NH (n = 10); untreated H patients experiencing a total bleeding ≥ 800 mL who did not receive any prohemostatic drugs group H (n = 4); and H patients experiencing a total bleeding of ≥ 800 mL receiving various doses of intravenous TA bolus since the onset of bleeding : group TA (n = 11). The patients’ characteristics are presented in Table 1. H patients were similar to NH except for CS duration and volume of bleeding as well as D-dimers and fibrinogen plasma level at T0.

**Table 1:** Pilot study patients clinical and biological profile

	Group NH (*n* = 10)	Group H + TA (*n* = 15)	*p*
Elective CS (%)	100	60	
Delay incision - inclusion (min)	16 [3]	31 [16]	0.008
CS duration (min)	48 [10]	98 [45]	0.002
Maternal age (years)	30.8 [5, 5]	31.8 [4, 4]	0.62
Total numer of gestations	2.8 [2, 7]	2.7 [1, 6]	0.92
Parity	2.6 [1, 2]	2.6 [1, 2]	0.95
Gestational age (SA)	37.5 [2, 7]	36.7 [3, 3]	0.52
Fetal weight (g)	3155 [1054]	2673 [966]	0.26
Bleeding volume (mL)	308 [138]	1501 [592]	<0.0001

#### TA concentration measurement method in venous and uterine blood and in urines

##### Technical performance and results

An analytical protocol based on liquid chromatography coupled with tandem mass spectrometry (LC-MS/MS) was developed, according to previously published methods [29], with slight modifications, to measure total TA concentration in EDTA plasma (venous and uterine blood) and in urine samples. Furthermore, a preliminary kinetic study on 11 TA-treated patients was performed to determine the optimal times for blood and urine sampling.

##### Chemicals and reagents

TA, 7β-hydroxyethyl-theophylline, methanol, and formic acid were purchased from Sigme Aldrich, Saint Quentin Fallavier, France. Fifty microliters of plasma or pre-diluted (1/10th) urine were mixed and centrifuged (4500 g, 4 °C, 10 min) after addition of 400 μL of methanol (for protein precipitation) containing 7β-hydroxyethyl-theophylline (Sigma-Aldrich, Saint Quentin Fallavier, France) at 20 mg/L, as an internal standard. The supernatant (20 μL) was added to water/formic acid 0.1% (180 μL). Five microliters of this mixture was injected into an UPLC-MS/MS system (Acquity Xevo-TQ Detector, Waters, Milford, MA, USA) equipped with a HSS T3 column (1.8 μm × 2.1 × 50 mm). Compounds were separated using a mobile phase gradient. Assay temperature was set up at 50 °C. Ions of each analyzed compound were detected in a positive ion mode using multiple reaction monitoring. Data acquisition and quantification were performed using MassLynx 4.1 Software (Waters). Calibration standards were obtained by dilution of the TA stock solution (100 g/L) at 200, 100, 50, 25, and 5 mg/L in water or blank plasma.

##### Results

Using TA-spiked plasmas, the limit of detection (LOD) and the lower limit of quantification (LLOQ) were determined at 1.2 mg/L and 2 mg/L, respectively. Linearity was tested up to 300 mg/L using the calibration range 5–200 mg/L (r^2^ = 0.995). Intra-day and inter-day precisions were < 3.80% and 5.30%, respectively, for a 20-mg/L-spiked sample and < 2.90% and 4.15%, respectively, for a 150-mg/L-spiked sample, which indicates that our method is highly precise and reproducible. The analysis of ten TA-free plasma samples and ten TA-free urine samples did not reveal any interference in TA detection, which indicates that our method is selective and specific for TA quantification.

A preliminary kinetic study was performed on 11 H patients receiving a unique IV dosage of 0.5, 1, or 2 g of TA in order to determine an average time-dependent profile of plasmatic concentrations of TA. This preliminary study (Fig. 2) highlighted that T1 (min), T15, T30, T60, T120, and T360 were appropriate times for blood sampling, regardless of the dosage used, to establish the TA plasmatic concentration curve. Urine collection at T120 and T360 (min) are also necessary to determine the amount (in mg) of excreted TA.

#### Simultaneous thrombin–plasmin generation assay

In 2011, van Geffen et al. developed a novel hemostasis assay which measures thrombin and plasmin generation in a single well by a fluorimeter [30]. By measuring the two key enzymes involved in coagulation, this method is the first to assess both clot formation and fibrinolysis and their interactions. The technique was slightly modified and set up in the Centre de Biologie Pathologie CHRU de Lille.

##### Chemicals and reagents

Thrombin and plasmin generation measurements were realized in a fluorimeter Fluoroskan Ascent® (Thermo-Labsystems, Helsinki, Finland). Black polystyrene 96 microtiter plates were purchased from Thermo-Labsystems as well. We used Tris Buffer Saline (TBS) 50 mM prepared with sodium chloride (Euromedex®), Trizma Base®, and Trizma hydrochloride® (Sigma), which was then filtered through a 0.8-μm Millex filter; calcium chloride 200 mM (VWR Prolabo®), filtered as well; recombinant activated tissue factor (TF) Innovin® (Siemens Healthcare Diagnostics, Marburg, Germany); cephalin CK®-Prest® (Diagnostica Stago); tissue-type plasminogen activator (t-PA) Alteplase® (Boehringer Ingelheim, Ingelheim am Rhein, Germany); pure human thrombin and human plasmin (Enzyme Research Laboratories, distributed by Kordia, Leiden, the Netherlands) used as calibrators; thrombin specific substrate Bz-β-Ala-Gly-Arg-7-amino-4-methylcoumarin and plasmin specific substrate bis-(CBZ-L-phenylalanyl-L-arginine amide)-rhodamine produced by Chiralix (Nijmegen, the Netherlands).

##### Technical performance and results

First, 80 μL of platelet-free plasma sample was added in every well. Then 110 μL cephalin was mixed with 110 μL TF (dilution 1:50). Then, 4 μL of this activator mixture was added in each well. The substrate mixture was prepared by mixing 205 μL thrombin substrate (final concentration 833 μM) with 102.5 μL plasmin substrate (final concentration 33 μM) and 6 μL of the substrate mixture was added in each well. A total of 10 μL TBS was added to a final volume of 100 μL. This mixture was narrowly mixed and preheated in the 37 °C thermostated fluorometer. In the meantime, the starting reagent was prepared, containing 4 μL t-PA diluted in 996 μL TBS (final concentration of 193 IU/mL) and 1000 μL CaCl_2_ (final concentration 16.7 mM).

After adding 20 μL of starting reagent and immediate shaking, the reaction began and fluorescence was measured every 30 s over 70 min. The thrombin- and plasmin-specific substrates had excitation wavelengths around 355 and 485 nm and the fluorescence emission was measured at 460 and 520 nm, respectively. The cumulative fluorescent signals were measured by Ascent Software and used to calculate in Microsoft Excel the first derivative of each value using this mathematical formula: Y’i = (Yi + 1 – Yi-1)/(Xi + 1 – Xi-1) (Yi = the y-axis value, i.e. the fluorescence intensity for a point i; Xi = the x-axis value, i.e. the time for the same point i). These first derivative curves represented the thrombin and plasmin generation rate curves. The first derivative values were then converted into thrombin and plasmin concentrations using a linear calibration curve. The two final curves represented the thrombin and plasmin generated concentration over time.

##### Parameters

To describe the proteolytic activity of both thrombin and plasmin, a number of parameters were defined and automatically calculated on a Microsoft Excel® macro program. A normal pooled plasma sample was used to illustrate them (Fig. 3).

For thrombin, four parameters were determined (Fig. 3):

1. Lag time thrombin generation (min), i.e. the time between initiation reaction and start of thrombin generation;
2. Thrombin peak time (min), i.e. the time when thrombin generation reached its maximal velocity;
3. Thrombin peak (nM), corresponding to the maximal velocity of thrombin production;
4. Area under the curve (AUC) (nM-min), i.e. the total amount of generated thrombin.

Three parameters were defined to explore plasmin generation (Fig. 3):

5. Plasmin peak (nM): the height of plasmin generation when the curve shifted from a convex to a linear shape and it represented the point of lysis of the clot by plasmin;
6. Fibrin lysis time (FLT) (min): the interval time between the plasmin peak time and the lag time;
7. Plasmin potential (nM-min), for the area under the curve during FLT, i.e. the amount of generated plasmin.

## Results

### In vitro test validation

A plasma pool from 100 patients with normal coagulation tests (i.e. PT > 80%, aPTT 30–35 s, and fibrinogen 2–4 g/L) was made and used in order to determine within run precision (intra-day) and intermediate precision (inter-day) for the simultaneous thrombin–plasmin generation assay (Tables 2 and 3). The intra-day coefficients of variation (CV %) presented in the tables above were < 10% for all thrombin and plasmin parameters and < 20% for inter-day assays.

**Table 2:** Intra-assay coefficient of variation (Variation coefficient (%)) for each parameter of the simultaneous thrombin–plasmin generation assay in a lab pool (*n* = 16)

	Thrombin				Plasmin		
Parameter studies	Lag time (min)	Peak time (min)	Peak (nM)	AUC (nM × min)	Peak (nM)	AUC (nM × min)	Lysis time (min)
Variation coefficient (%)	8.42	8.59	6.18	1.90	8.70	5.08	5.46

**Table 3:** Inter-assay coefficient of variation (Variation coefficient (%)) for each parameter of the simultaneous thrombin–plasmin generation assay in a lab pool (*n* = 16)

	Thrombin				Plasmin		
Parameter studies	Lag time (min)	Peak time (min)	Peak (nM)	AUC (nM × min)	Peak (nM)	AUC (nM × min)	Lysis time (min)
Variation coefficient (%)	13.69	10.91	9.35	8.56	20.90	14.35	19.39

The next step was to evaluate the effect of TA on the STPGA profiles both in vitro and in vivo. To evaluate the in vitro effect of TA on STPGA, spiking was realized in a normal pooled plasma to obtain a final TA plasmatic concentration in each well in the range of 0–20 mg/L. Whereas thrombin generation was unaffected (Fig. 4a), plasmin generation decreased with rising TA concentrations (Fig. 4b). In vitro, STPGA showed the dose effect relationship between TA and plasmin generation (Fig. 4b).

### In vivo application

STPGA test was sampled at the different observation times in 15 haemorrhagic patients treated or not by various doses of TA. STPGA was described at T30 for thrombin and plasmin regarding the increased doses of TA (Fig. 5a and b, respectively). Then STPGA evolution over time curves were described in each group for thrombin (Fig. 6A-0, A-1/2, A-1, A-2, A-NH) and plasmin (Fig. 6B-0, B-1/2, B-1, B-2, B-NH).

In this pilot study, TA had no or very low influence on thrombin generation whereas plasmin generation seems dose-dependent.

Thrombin and plasmin generation at T0 in the NH, H, and TA groups were compared to the NP group (Fig. 7a and b).

Inter-individual dispersion of plasmin AUC was higher in the NH and H + TA groups than in the NP group at T0. Regarding inter-assay variation coefficient of the STPGA (<20% for all parameters) and evolution of STPGA profiles over time, inter-individual dispersion was compatible with the STPGA use for TRACES trial.

### Dose ranging simultaneous variations of plasmin AUC and TA concentration

**Fig. 8:**
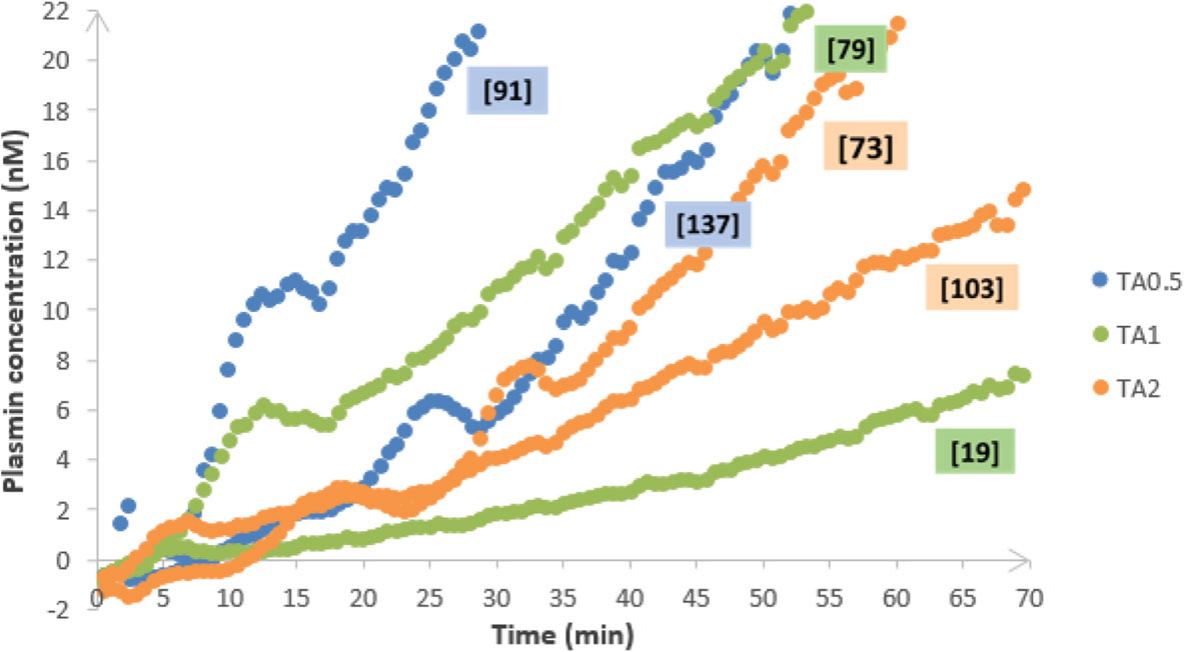
Plasmin generation profile at T0 (baseline) and corresponding TA peak plasma concentration at T1 (following injection) in TA-treated patients. Plasmin generation profiles at T0 (baseline, before injection) and corresponding TA peak plasma concentrations at T1 (just after injection, values in mg/L bracketed in little sidebars) of six patients treated by TA: two patients by dose (0.5, 1, and 2 g)

**Fig. 9:**
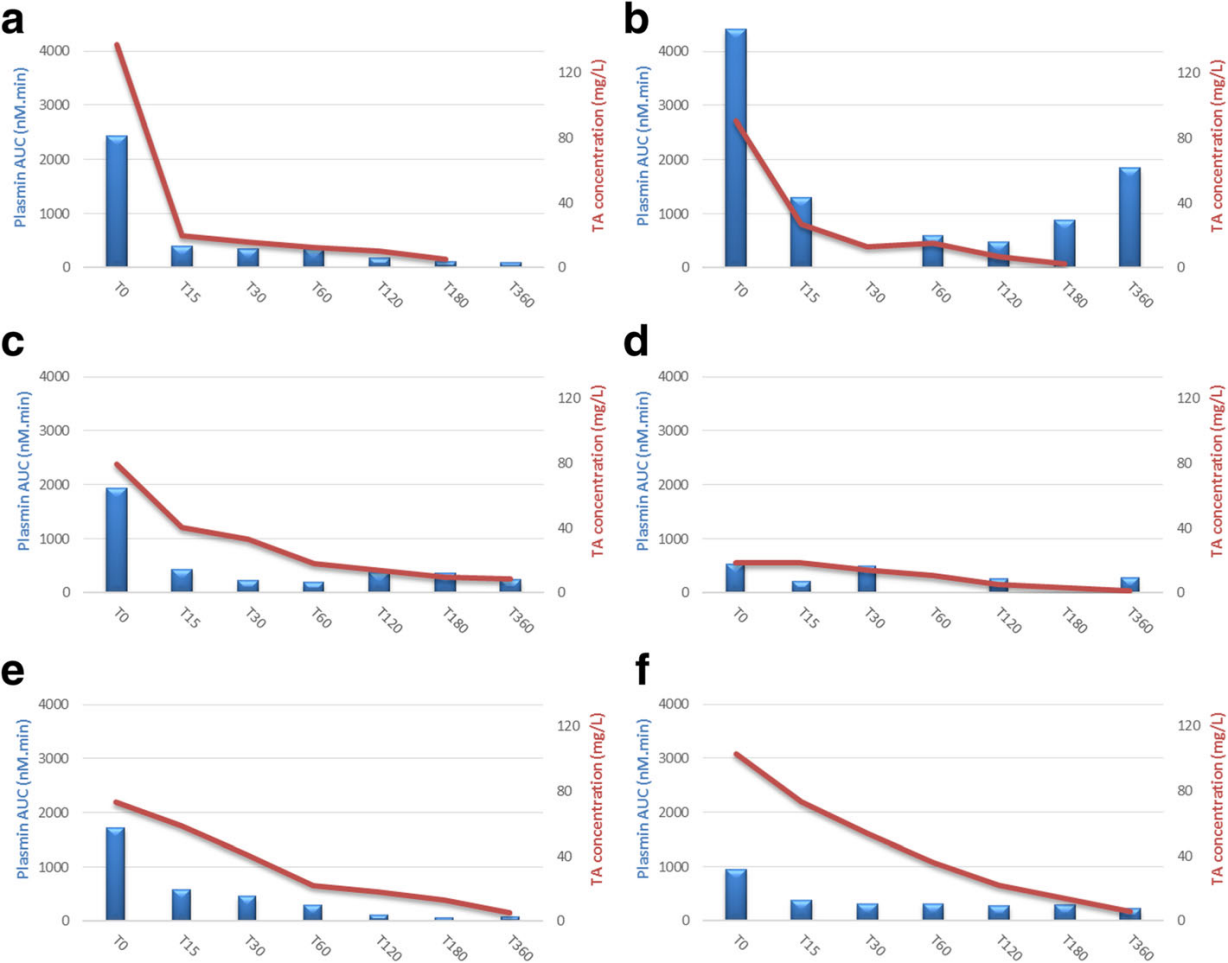
Kinetics of both TA plasma concentration and plasmin AUC in TA-treated patients: evolution of TA plasma concentration and plasmin AUC over time points: T0, T15, T30, T60, T120, T180, and T360, in six patients treated by TA (0.5, 1, or 2 g). **a** Kinetics of TA plasma concentration and plasmin AUC: group TA0.5 - patient 20. **b** Kinetics of TA plasma concentration and plasmin AUC: group TA0.5 - patient 19. **c** Kinetics of TA plasma concentration and plasmin AUC: group TA1 - patient 24. **d** Kinetics of TA plasma concentration and plasmin AUC: group TA1 - patient 25. **e** Kinetics of TA plasma concentration and plasmin AUC: group TA2 - patient 12. **f** Kinetics of TA plasma concentration and plasmin AUC: group TA2 - patient 17

The simultaneous variation of plasmin AUC and TA concentrations are presented in Figs. 8 and 9 in a patient receiving TA at the dose of 0.5 g (Fig. 9a and b), 1 g (Fig. 9c and g. d), or 2 g (Fig. 9e and f), respectively.

Plasmin AUC decreased in all cases 15 min after TA administration and its inhibition persisted, even though TA concentration decreased at a minimal level < 10 mg/L after 6 h (T360). However, plasmin increased again (or rose again) since T180 in one patient who had a large amount of plasmin generation at T0 (plasmin AUC > 4000 nM*min) and received 0.5 g of TA.

These observations concerning the possible variations of the expected effect of a single TA administration regarding the fibrinolytic intensity highlight the need of the TRACES study and its pharmacobiological ancillary substudy to determine the optimal dose, better timing, and the need for repeated doses or continuous infusion in H CS.

## Study organization

The coordinating team includes the coordinator, the promotor, and the major investigators of each of the centers. A call conference will be organized every two months between the coordinator and the centers, supported by a newsletter reporting inclusion rates and protocol information. Data and consent collection will be monitored by the promotor. An independent safety monitoring committee is recruited to observe blinding safety issues and allow the trial continuation. Data management and statistics will be done by an independent unit. Plans are written in advance for investigators and the sponsor to communicate trial results to participants, healthcare professionals, the public, and other relevant groups (e.g. via publication, reporting in results databases, or other data sharing arrangements).

## Trial status

TRACES inclusions have already begun. In the first opened center (promoter center), 79 patients have been included (59 H and 7 NH). The four other centers are opened and included 13 patients. The pharmacobiological substudy status is followed: complete TRACES-specific sampling has been in 60 out of the 79 inclusions. Three monitoring committee meetings monitored these initial data and allowed the pursuit of the trial.

## Additional files

Additional file 1: SPIRIT 2013 Checklist: Recommended items to address in a clinical trial protocol and related documents*. (DOC 131 kb)

Additional file 2: Schedule of enrolment, interventions and assessments. (DOC 44 kb)
