# Supplementary material for: TRAnexamic acid in hemorrhagic CESarean section (TRACES) randomized placebo controlled dose-ranging pharmacobiological ancillary trial: study protocol for a randomized controlled trial
Source: Trials. 2018 Mar 1;19:149. doi: 10.1186/s13063-017-2421-6 (PMC5831219; doi:10.1186/s13063-017-2421-6)
Supplement: Supplementary file 2 — Schedule of enrolment, interventions and assessments. (DOC 44 kb) [file 13063_2017_2421_MOESM2_ESM.doc]

Spirit Figure : Schedule of enrolment, interventions, and assessments.

|  |  |  |  | Study period | | | | | | | | | |  |
| --- | --- | --- | --- | --- | --- | --- | --- | --- | --- | --- | --- | --- | --- | --- |
|  | Enrolment | | Allocation | |  |  |  | Post-allocation | | | | | Close out |  |
| Timepoint | T-1 | | T0:T1 | | T15 | T30 | T60 | T120 | T180 | T360 | D2 | D42 |  | |
| Enrolment | x | |  | |  |  |  |  |  |  |  |  |  | |
| Elligibility screen | x | |  | |  |  |  |  |  |  |  |  |  | |
| Informed consent | x | |  | |  |  |  |  |  |  |  |  |  | |
| Availability of biological samples conditioning for specific samples | x | |  | |  |  |  |  |  |  |  |  |  | |
| Allocation |  | | TA current dose  0 to 2g | |  |  |  |  |  |  |  |  |  | |
| INTERVENTIONS |  |  |  | | | | | | | | | | | |
| TRACES biological sampling: current hemoglobin fibrinogen DDimers | x | |  | |  |  |  |  |  | x | x |  |  | |
| TRACES specific biological sampling: TA concentration and plasmin generation |  | | x | |  | X | X | X | X | x |  |  |  | |
| Assessments |  | |  | |  |  |  |  |  |  |  |  |  | |
| Baseline | x | | x | |  |  |  |  |  |  |  |  |  | |
| TRACES Primary endpoint:  Blood loss measurement | x | |  | |  |  |  | x |  | x |  |  |  | |
| Safety endpoints |  | |  | |  | X | X | X | X |  | X | X | x | |
| Data monitoring and management |  | |  | |  |  |  |  |  |  |  |  | x | |
